# Carcinosarcoma of the Parotid With Osteosarcoma Component: A Case Report

**DOI:** 10.1002/ccr3.71902

**Published:** 2026-02-04

**Authors:** Shubham Dokania, Ajay S. Krishnan, Ashutosh Mukherji, Pritam Mondal, Ipsita Dhal, Zachariah Chowdhury, Shreya Shukla, Akhil Kapoor, Sunayana R. Sarkar

**Affiliations:** ^1^ Department of Radiation Oncology Mahamana Pandit Madan Mohan Malaviya Cancer Centre Varanasi India; ^2^ Department of Radiation Oncology Mahamana Pandit Madan Mohan Malaviya Cancer Centre, Varanasi, Homi Bhabha National Institue Varanasi India; ^3^ Department of Oncopathology Mahamana Pandit Madan Mohan Malaviya Cancer Centre, Varanasi, Homi Bhabha National Institue Varanasi India; ^4^ Department of Radiodiagnosis Mahamana Pandit Madan Mohan Malaviya Cancer Centre, Varanasi, Homi Bhabha National Institue Varanasi India; ^5^ Department of Medical Oncology Mahamana Pandit Madan Mohan Malaviya Cancer Centre, Varanasi, Homi Bhabha National Institue Varanasi India; ^6^ Department of Surgical Oncology Mahamana Pandit Madan Mohan Malaviya Cancer Centre, Varanasi, Homi Bhabha National Institue Varanasi India

**Keywords:** carcinosarcoma, chemoradiotherapy, osteosarcoma, parotid

## Abstract

Carcinosarcomas of the salivary gland with osteosarcoma component are very rare, with only 17 cases reported. Despite multiple poor prognostic factors, our patient's outcome was favorable, highlighting the importance of early diagnosis and aggressive treatment in improving prognosis for this aggressive tumor variant.

## 1 Introduction

Carcinosarcoma is a rare and aggressive malignant mixed tumor of the salivary gland, alongside carcinoma ex pleomorphic adenoma and metastasizing pleomorphic adenomas. These tumors contain both epithelial (carcinomatous) and mesenchymal (sarcomatous) components. Carcinosarcomas account for 0.04%–0.16% of all salivary gland tumors and 0.4% of salivary gland malignancies, predominantly affecting the parotid gland. The carcinomatous component is usually squamous cell carcinoma (SCC) or adenocarcinoma. The sarcomatous counterparts can be chondrosarcoma, fibrosarcoma, leiomyosarcoma, osteosarcoma, and liposarcoma, in decreasing frequency [1]. Although carcinosarcomas can arise de novo, about 30% occur in patients with longstanding or recurrent pleomorphic adenomas, known as carcinosarcoma ex pleomorphic adenoma. The lungs, bones, and central nervous system are the most frequent sites of metastasis [2].

Fewer than 200 cases of salivary gland carcinosarcoma have been documented in the literature since its first description by Kirklin et al. in 1951 [3]. Carcinosarcoma with osteosarcoma as a sarcomatous component is even rarer, with only 17 reported cases. We present a case of de novo carcinosarcoma of the left parotid gland, comprising adenocarcinoma and osteosarcoma components. The patient underwent parotidectomy followed by adjuvant chemoradiotherapy (CTRT), demonstrating a significantly better outcome than most previously reported cases.

## 2 Case History/Examination

A 73‐year‐old male with a 50‐year history of chewing tobacco and no known comorbidities presented to us in January 2022 with left‐sided facial swelling. It was gradually progressive in size for the last 7 months. There was no associated pain, dysphagia, loss of taste sensation, ear‐related symptoms, or trismus. On examination, he had a Karnofsky Performance Score (KPS) of 90. A firm, non‐tender, and non‐fluctuant 7*5 cm mass was found over the left parotid region. There was no rise in local temperature, no signs of facial nerve palsy, or any palpable cervical nodes.

## 3 Methods (Differential Diagnosis, Investigations and Treatment)

After a thorough history and physical examination, we had limited differential diagnoses, namely chronic abscess, granulomatous, or neoplastic involvement. MRI of the face and neck showed a 5.2*4.8*4.7 cm lesion over the left side of the face involving both the deep and superficial lobes of the parotid and abutting the ipsilateral sternocleidomastoid and medial pterygoid muscles. Fine needle aspiration cytology (FNAC) confirmed malignancy, with the likely possibilities being salivary duct carcinoma and high‐grade mucoepidermoid carcinoma. The patient was staged as cT3N0M0 (as per AJCC 8th edition) and planned for radical parotidectomy.

**FIGURE 1:**
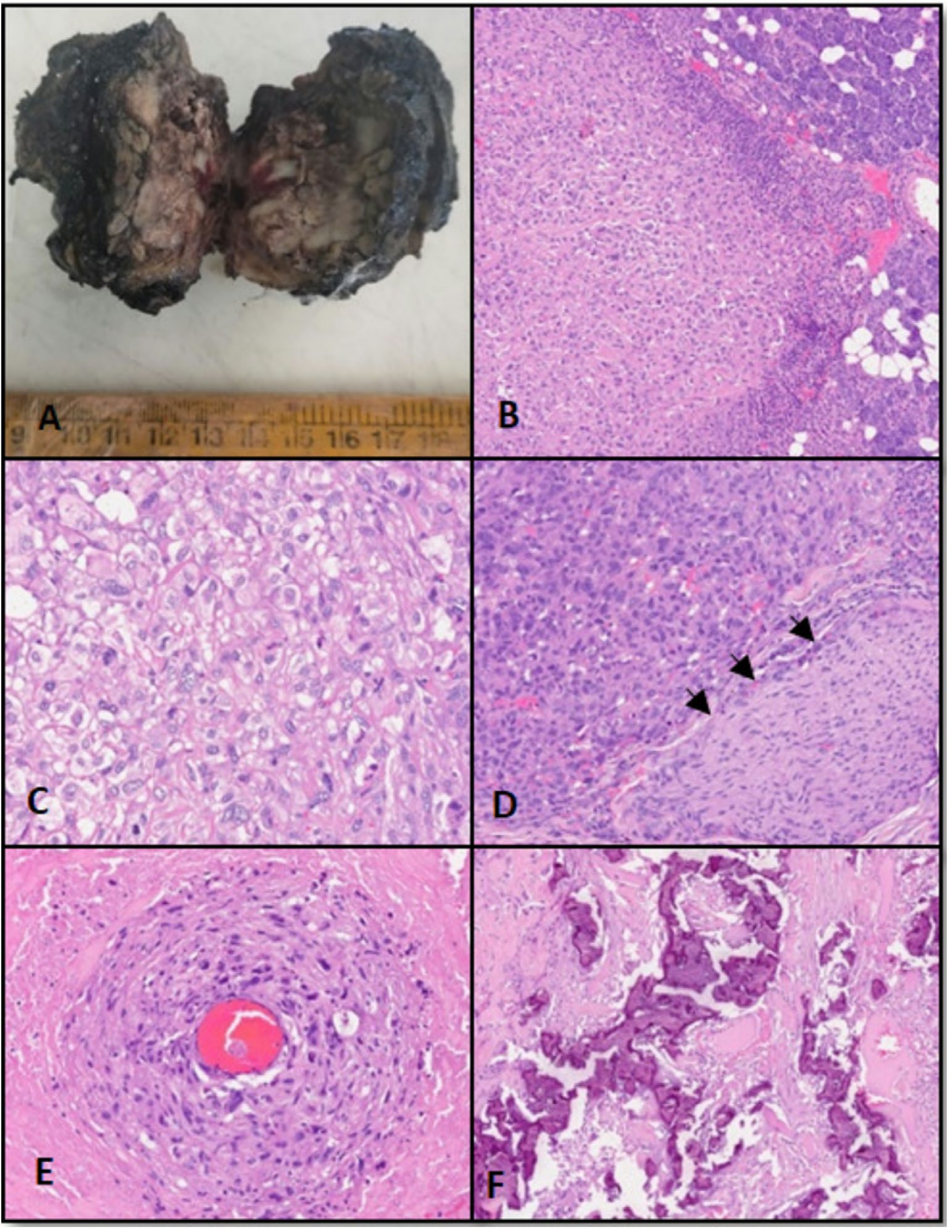
(A) Gross image of cut section of the parotidectomy specimen shows a solid tumor with variegated appearance measuring 4.5 × 3 × 3.5 cm. (B) Microsection (H and E, 10X) shows an infiltrating tumor along with adjacent normal salivary gland acini. (C) Diffuse sheeting of the tumor cells showing clear cell morphology (H and E, 40X). (D) Tumor showing perineural invasion (arrows). (E) Perivascular accentuation of the tumor cells is noted. (F) Extensive areas of calcification are seen.

**FIGURE 2:**
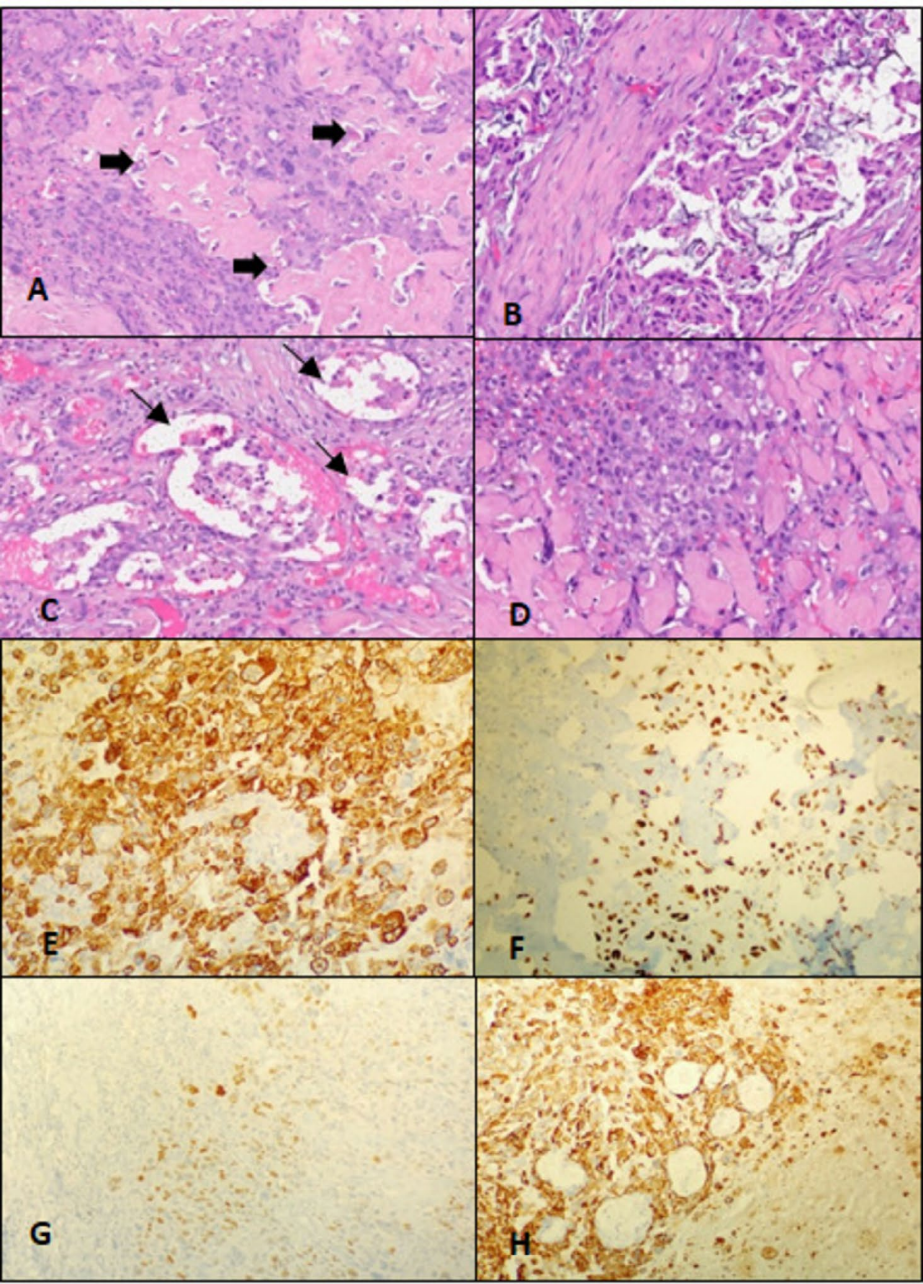
(A) Osteoid formation by the tumor cells is seen (arrows). (B) Microsection (H and E, 40X) shows glandular pattern of arrangement of tumor cells with large areas of extracellular mucin. (C) Vascular invasion by the tumor cells is noted (arrows). (D) Tumor cells are infiltrating the skeletal muscle bundles. (E) Diffuse positivity of the epithelial component of tumor cells by PanCK immunohistochemistry. (F) SATB2 is focally positive. (G) Focal positivity for p40 is seen. (H) CK7 is strongly positive in the glandular components.

He underwent left radical parotidectomy, left Type III modified neck dissection (Ia, left Ib‐IV, Va, and Vb), sternocleidomastoid rotation flap, and tarsorrhaphy with facial nerve sacrifice in February 2022. The intraoperative and postoperative course was uneventful. The postoperative histopathological report revealed de novo carcinosarcoma with distinct carcinomatous and sarcomatous components. It was a solid tumor of size 4.5 × 3 × 3.5 cm, with a variegated appearance (Figure 1A), involving both lobes of the left parotid gland. Focal lymphovascular invasion (LVI) (Figure 2C), perineural invasion (Figure 1D), and skeletal muscle infiltration (Figure 2D) were present along with a positive superior resection margin, but the facial nerve and skin were free. The carcinomatous component showed a glandular pattern of arrangement of tumor cells with large areas of extracellular mucin, suggesting poorly differentiated adenocarcinoma (Figure 2B). Also, there were highly pleomorphic tumor cells with a high N/C ratio, irregular nuclei, vesicular chromatin, prominent nucleoli, and diffuse sheeting of the tumor cells showing clear cell morphology (Figure 1C). The sarcomatous component was heterologous and composed of malignant osteoid (Figure 2A) along with bone formation and extensive areas of calcification (Figure 1F). Other histological features have been illustrated in Figure 1B,E. Out of 44 dissected neck nodes, none came positive, and it was staged pathologically as pT3pN0 (as per AJCC 8th edition).

On immunohistochemistry (IHC), the adenocarcinoma component was highlighted by the presence of AE1/AE3 (panCK) (Figure 2E) and CK7 (strong and membranous) (Figure 2H); the osteosarcoma component by SATB2 (strong and nuclear) (Figure 2F). Focal positivity for p40 (Figure 2G) and p63 was also seen. The FNAC suspicion of salivary duct carcinoma was ruled out by the absence of GATA 3, androgen receptor, and Cerb B2 in IHC. pT3 stage, positive margins, LVI, and PNI led to a decision for adjuvant CTRT to the post‐operative bed. The decision regarding concurrent chemotherapy was taken after discussion in a tumor board, as there was no level 1 evidence for the same. Cisplatin was deferred due to grade 3 sensorineural hearing loss (SNHL) in the left ear, and weekly paclitaxel 80 mg/m^2^ and carboplatin (AUC 2) were planned.

We delivered Radiotherapy (RT) to the tumor bed and dissected nodal stations to a dose of 54 Gy in 30 fractions (1.8 Gy per fraction). A simultaneous integrated boost (SIB) up to 66 Gy was delivered to the tumor bed. Daily fractions, 5 days a week, were delivered by volumetric modulated arc technique (VMAT) along with six cycles of concurrent paclitaxel and carboplatin. There was a gap of 18 days during RT, due to radiation therapy oncology group (RTOG) grade 3 skin toxicity; apart from this, there was no grade 3 or above acute toxicity (mucositis, pharyngitis). The skin toxicity was managed conservatively. Apart from these, there were grade 2 neutropenia and thrombocytopenia, which were resolved within 1 week of treatment completion. The patient was kept on a 3‐monthly follow‐up. In the 1st year of follow‐up, he developed grade 1 subcutaneous fibrosis of neck, which resolved gradually with neck physiotherapy. Grade 1 xerostomia resolved spontaneously by the end of 1 year.

## 4 Conclusions and Results (Outcome and Follow‐Up)

**FIGURE 3:**
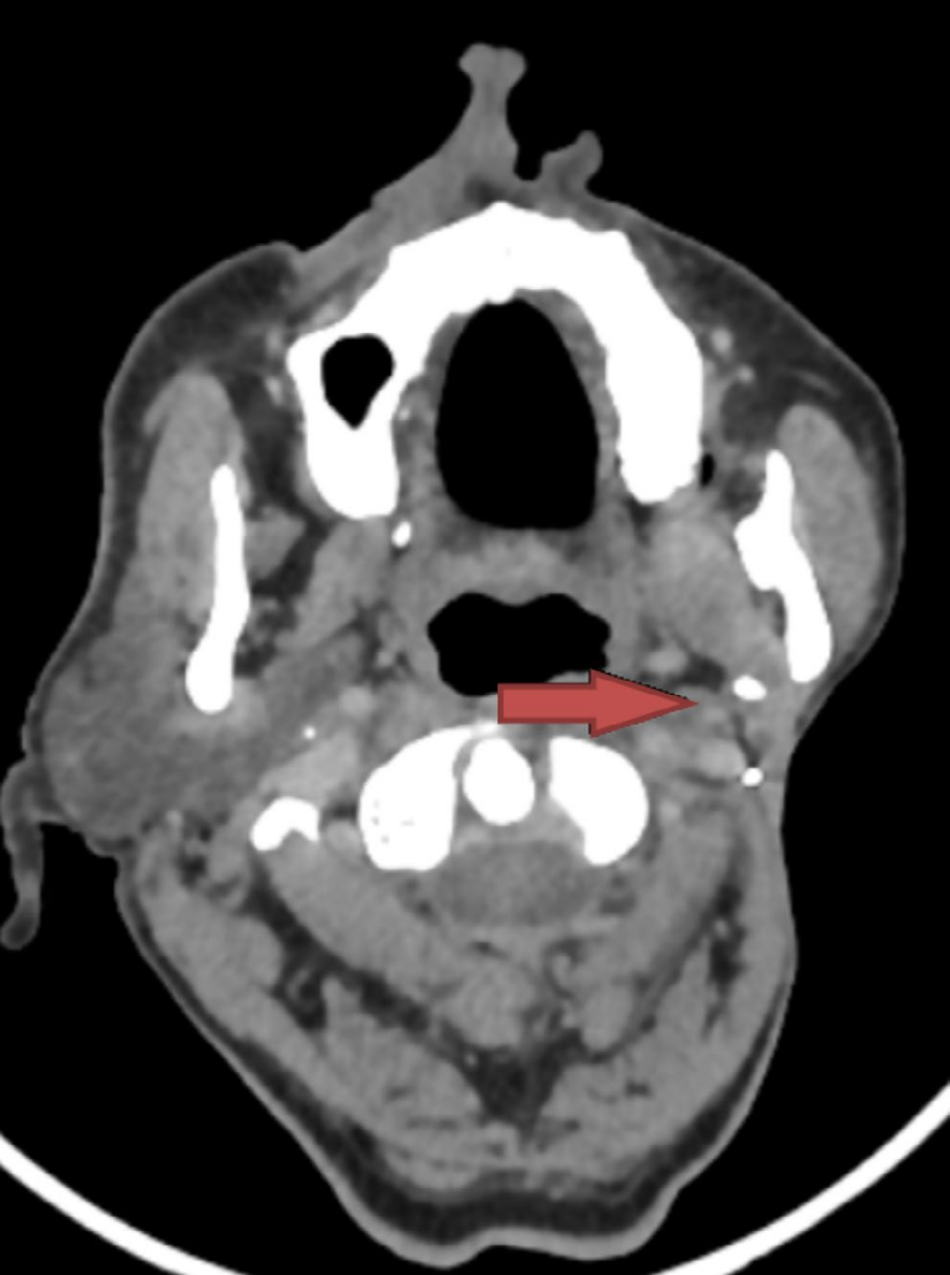
Post left parotidectomy status, post 2.5 years of adjuvant therapy completion. Red arrow indicated the post‐parotidectomy bed.

The patient is on regular follow‐up and is free from any symptoms. The latest CECT face and neck, done after 2.5 years of completion of adjuvant therapy, showed no evidence of disease recurrence, as shown in Figure 3. After more than 3 years of treatment completion, the patient has no local, regional, or distant relapse clinicoradiologically.

## 5 Discussion

Carcinosarcoma histology has been demonstrated in multiple organs apart from salivary glands, such as the uterus, bladder, and lungs [4]. The average age at which individuals present typically falls within the sixth to seventh decade of life, although it can span from 14 to 87 years. Multiple hypotheses have been proposed regarding its pathogenesis [5, 6]. Based on pathology in various body systems, four main theories have been proposed [7]. The collision tumor theory posits that two separate tumors have merged, often seen with skin cancers and malignant fibrous histiocytomas in sun‐damaged skin. The composition theory views the mesenchymal part as a reaction to the epithelial cancer. The combination theory suggests that both cancer types originate from the same stem cell but differentiate differently. The conversion/divergence theory proposes that the sarcomatous component evolves from the epithelial one by metaplastic change. Studies in IHC and molecular genetics lean towards a monoclonal origin for carcinosarcoma.

Salivary carcinosarcoma is managed with surgery, which is usually followed by adjuvant RT, considering its high grade. However, even with this treatment, as per historical data, more than half of patients succumb to either local recurrence or metastasis. The mean survival of these patients is 3.6 years [8, 9]. Even recent retrospective reviews show a similar picture. Surveillance, Epidemiology, and End Results (SEER) database‐based analysis of 66 patients of carcinosarcoma of salivary glands by Gupta et al. showed the median survival to be 38 months. Overall survival (OS) was 68.1% and 37.2% at 2 and 5 years, respectively, with a distant metastases rate of 17.2%. Disease‐specific survival was 78.3% and 62.2% at 2‐ and 5‐year intervals, respectively. Univariate analysis showed that out of multiple factors, only distant metastases and total parotidectomy significantly affected mortality rates [10]. Another National Cancer Database (NCDB)‐based review of 154 patients by Talwar et al. is supposedly the largest retrospective study on this topic. Only 108 patients met the inclusion criteria for survival analysis, which revealed two‐year and three‐year OS to be 62.8% and 57.6%, respectively. Univariate analysis showed higher mortality risk for advanced age, pT3‐4 stage, presence of nodal metastases (pN+), and positive surgical margins. Only age and pT3/4 disease were found to be significant in multivariate analysis [11]. None of these studies stratified their survival analysis according to the histology of carcinomatous or sarcomatous components. Surgery followed by adjuvant RT was the treatment used in 76.8% and 65% patients in the above‐mentioned studies, respectively. Chemotherapy in the adjuvant setting, with or without concomitant RT, was used only in 17%–18% cases in either study.

We did a literature review on PubMed and Google Scholar using the keywords: Osteosarcoma AND carcinosarcoma. Only 17 cases of salivary gland carcinosarcoma with osteosarcoma as a sarcomatous component could be found [6, 8, 12, 13, 14, 15, 16, 17, 18, 19, 20, 21, 22, 23, 24, 25]. We analyzed these 18 cases (including our patient); their clinicopathological features and outcomes have been tabulated along with the present case in Table 1. Our analyses showed a higher proportion of males (72.3%), with age ranging from 35 to 83 years (mean age‐ 62.3 years and median age‐ 64 years). Among 18 cases, carcinomatous components were adenocarcinoma in 10 (55.5%), adenosquamous in four (22.2%), salivary duct carcinoma in three (16.6%), and mucoepidermoid carcinoma in one (6.6%) case. Eight cases (44.4%) had mixed sarcomatous components in addition to osteosarcoma; fibrosarcoma in two, and chondrosarcoma in seven of them; three of them even had more than two sarcomatous components. The observation of multiple sarcomatous components in the same patient lends more credence to the combination hypothesis over the composition hypothesis of pathogenesis.

**TABLE 1:** Clinicopathological features and outcomes of the reported cases of Carcinosarcoma salivary glands with osteosarcoma component.

References	Age (years)/sex	Site	Histologic type of carcinoma	Histologic type of sarcoma	Treatment	Outcome
Garner et al. [8]	57/F	Parotid gland	Adenocarcinoma	Osteosarcoma, chondrosarcoma	Left parotidectomy and radical neck dissection	Local failure at 6 weeks
Yamashita et al. [16]	52/M	SMG	Adenosquamous carcinoma	Osteosarcoma, chondrosarcoma, fibrosarcoma	Excision and right modified neck dissection with adjuvant chemotherapy	No recurrence for 5 months
Bleiweiss et al. [17]	64/M	SMG	Adenocarcinoma	Osteosarcoma	Partial mandibulectomy with radical neck dissection	Local failure at 4 months
de la Torre et al. [18]	83/M	Parotid gland	Adenocarcinoma	Osteosarcoma, chondrosarcoma	Wide excision	Local failure and lung metastasis within 1 year
Carson et al. [19]	51/F	Parotid gland	Adenocarcinoma	Osteosarcoma	Total parotidectomy with adjuvant chemotherapy	Local failure at 7 months
Gogas et al. [15]	77/M	SMG	Salivary duct carcinoma	Osteosarcoma, chondrosarcoma, rhabdomyosarcoma	Wide excision with adjuvant CTRT	Lung metastasis at 3 months
Sironi et al. [20]	77/M	Parotid gland	Adenosquamous carcinoma	Osteosarcoma	Right total parotidectomy with radical neck dissection	Metastasis at 3 months
Mardi et al. [21]	59/M	Parotid gland	Adenocarcinoma	Osteosarcoma, chondrosarcoma	NA	NA
Staffieri et al. [14]	46/F	Parotid gland	Adenocarcinoma	Osteosarcoma, chondrosarcoma	Total parotidectomy with CTRT	No recurrence at 26 months
Yura et al. [24]	54/M	SMG	Adenosquamous carcinoma	chondrosarcoma, osteosarcoma, spindle cell sarcoma, rhabdomyosarcoma, and liposarcoma	Wide excision and neck dissection with adjuvant CTRT	No recurrence at 24 months
Jang et al. [22]	67/M	Parotid gland	Adenocarcinoma	Osteosarcoma	Left parotidectomy with adjuvant RT	Lung metastasis at 5 months
Feng et al. [26]	76/F	Parotid gland	Salivary duct carcinoma	Osteosarcoma, fibrosarcoma	Parotidectomy alone	No recurrence at 12 months
Jha et al. [6]	35/M	Parotid gland	Adenocarcinoma	Osteosarcoma	Total radical parotidectomy with adjuvant RT	No recurrence for 12 months
Woo et al. [12]	72/M	Parotid gland	Adenosquamous carcinoma	Osteosarcoma	Total parotidectomy with adjuvant RT	Abdominal Metastasis at 6 months
Endo et al. [25]	64/M	SMG	Adenocarcinoma	Osteosarcoma	Wide excision and neck dissection with adjuvant CTRT	Lung Metastasis at 4 months
Kwon et al. [13]	64/F	Parotid gland	Mucoepidermoid carcinoma	Osteosarcoma	Total parotidectomy with selective neck dissection with adjuvant CTRT	No recurrence for 18 months
Tang et al. [23]	45/M	Parotid gland	Salivary duct carcinoma	Osteosarcoma	Total parotidectomy with CTRT	No recurrence for 12 months
Present case	73/M	Parotid gland	Adenocarcinoma	Osteosarcoma	Total parotidectomy with adjuvant CTRT	No recurrence for 38 months

Thirteen cases (72.2%) involved the parotid gland and five (27.7%) involved the submandibular gland (SMG). Nine out of 18 (50%) cases failed locally or distally within 1 year of treatment completion; treatment details, as well as outcomes of one could not be found. Out of these nine patients, four (22%) failed locally, six (33.3%) failed systemically, and one (5.5%) failed both locally and distally. We find that the median age of those failing systemically (72 years) is higher than for those failing locally (57.5 years), which in turn is higher than for those who had no recurrence (53 years). We find these results consistent with the finding that older age is a poor prognostic factor in Carcinosarcomas.

Among the four patients failing locally, only one was elderly; three patients had disease in the parotid, and one had disease in the SMG. Two underwent total parotidectomy, one had partial mandibulectomy for SMG tumor, and one had only a wide excision. Only one received chemotherapy, and none received RT. The elderly patient was the one who had local excision, without chemotherapy or RT, and developed both local and distant recurrence within a year. Chi‐square analysis reveals that omitting RT is correlated with local recurrence (*p* = 0.006).

Out of the five patients with isolated distant metastases, three were of parotid and two were of SMG origin, with four of them aged more than 65 years. Three patients did not receive chemotherapy. One who received CTRT was elderly with an adverse histology‐ salivary duct carcinoma, which could have contributed to the development of metastases despite CTRT. Another one who received CTRT received S‐1 (Tegafur, Gimeracil, Oteracil) chemotherapy concurrently, which is not standard for Head and Neck cancers or carcinosarcomas. A trend of increased distant metastases is observed with avoiding chemotherapy, but is not substantiated by the chi‐square test.

Among the eight patients free of recurrence, only two were elderly. All had adequate surgeries done; six had total parotidectomy, and two had wide excision of SMG tumor. Two received adjuvant chemotherapy alone, one got adjuvant RT alone, and four were treated with adjuvant CTRT, while one of them received no adjuvant treatment.

Our analysis shows that carcinosarcoma with osteosarcoma component has poorer outcomes. Our patient comes under high risk of mortality from the disease due to both higher age and stage, apart from having margin‐positive resection, focal LVI, and PNI. Yet he is disease‐free even after 3 years of treatment. The possible reasons for a better outcome in our case include radical resection, tumor bed RT boost to 66 Gy (due to positive margin), and the use of concurrent chemotherapy with an effective regimen. For parotid malignancies in general, there is no level 1 evidence for the use of concurrent chemotherapy with adjuvant RT. A large retrospective study by Amini et al. failed to show any OS benefit with concurrent chemotherapy [27]. RTOG 1008 results seeking any progression‐free survival (PFS) benefit with concurrent chemotherapy are underway [28]. Since our elderly case had multiple poor prognostic factors along with osteosarcoma component, we had to select an effective as well as less toxic concurrent chemotherapy regimen. Cisplatin was deferred as our patient had grade 3 SNHL in the left ear. We preferred paclitaxel‐carboplatin over carboplatin‐5FU, as the former has been proven to be as effective as and less toxic than the latter in several phase II studies [29, 30]. The strengths of our report include a new addition to a small repertoire of 17 rare cases and the availability of a long follow‐up of more than 3 years. A chi‐square correlation between the prognostic factors and the outcomes also adds to the strengths. The lack of information about the long‐term outcomes of the other similar cases is a limitation of our study.

In essence, post‐total parotidectomy, adjuvant treatment appears to be necessary in carcinosarcomas with osteosarcoma component. Considering the propensity for distant metastasis without chemotherapy and for local failures without RT, adjuvant CTRT is likely a reliable option in carcinosarcomas with osteosarcoma component. Advanced age appears to be a significant poor prognostic factor, but adjuvant CTRT may even offset its adverse effect. IHC has a significant role in histological diagnosis. Further studies are needed to evaluate the contribution of different histologies to the outcomes of the patient.

## Author Contributions

**Shubham Dokania:** writing – original draft. **Ajay S. Krishnan:** writing – original draft, writing – review and editing. **Ashutosh Mukherji:** writing – review and editing. **Pritam Mondal:** writing – review and editing. **Ipsita Dhal:** resources. **Zachariah Chowdhury:** resources. **Shreya Shukla:** resources. **Akhil Kapoor:** writing – review and editing. **Sunayana R. Sarkar:** writing – review and editing.

## Funding

The authors have nothing to report.

## Ethics Statement

The authors have nothing to report.

## Consent

Written informed consent was obtained from the patient to publish this report in accordance with the journal's patient consent policy.

## Conflicts of Interest

The authors declare no conflicts of interest.

## Data Availability

The data that support the findings of this study are available on request from the corresponding author. The data are not publicly available due to privacy or ethical restrictions.
